# Full Thermal Switching of Enzymes by Thermoresponsive Poly(2‐oxazoline)‐Based Enzyme Inhibitors

**DOI:** 10.1002/chem.202001909

**Published:** 2020-09-23

**Authors:** Montasser Hijazi, Esra Türkmen, Joerg C. Tiller

**Affiliations:** ^1^ Department of Bio- and Chemical Engineering TU Dortmund Emil-Figge-Str. 66 44227 Dortmund Germany

**Keywords:** enzyme inhibitors, functional polymer end groups, lower critical solution temperature polymers, poly(2-oxazoline), thermoresponsive

## Abstract

Controlling the activity of enzymes is an important feature for many processes in medicine, bioanalytics, and biotechnology. So far, it has not been possible to fully switch biocatalysts on and off by thermoresponsive enzyme inhibitors. Herein, we present poly(2‐oxazoline)s with iminodiacetic acid end groups (POx‐IDA) that are lower critical solution temperature (LCST) polymers and thus thermosensitive. They are capable of reversibly inhibiting the activity of horse radish peroxidase and laccase by more than 99 %. Increasing the temperature makes the POx‐IDA precipitate, which leads to 100 % recovery of the enzyme activity. This switching cycle is fully reversible. The LCST of the POx‐IDA can be tuned by varying the polymer composition to generate a wide range of switching windows.

## Graphical abstract

**Thermoresponsive co(poly‐2‐oxazoline)s** with a 2,2′‐imino diacetate end group are capable of fully deactivating the enzymes laccase and horse radish peroxidase at different temperatures varying from 4–40 °C. Increasing the temperature results in full activity of both enzymes due to aggregation of the polymeric inhibitors. The process is fully reversible and does not interfere with the protein′s activity.

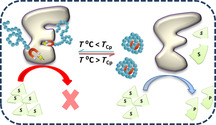

## Introduction

Controlling the activity of enzymes is a key issue for influencing the biochemistry of life‐forms, required in medicine and biotechnology, but it is also useful for varying the activity of biosensors. For example, many processes such as measuring metabolites in blood or determining certain oxidizing compounds in water are based on enzymatic assays, which measure a signal increase with time affording a precise preparation time and punctual measurement. Controlling the activity of the enzyme would greatly improve the reliability of such crucial measurements by switching the respective enzyme on only for a certain period of time and switching it off afterwards. In terms of medical treatment it would be of great benefit to reversibly control the in vivo activity of drugs, which are very often enzyme inhibitors, by using external triggers.

**Figure 1:**
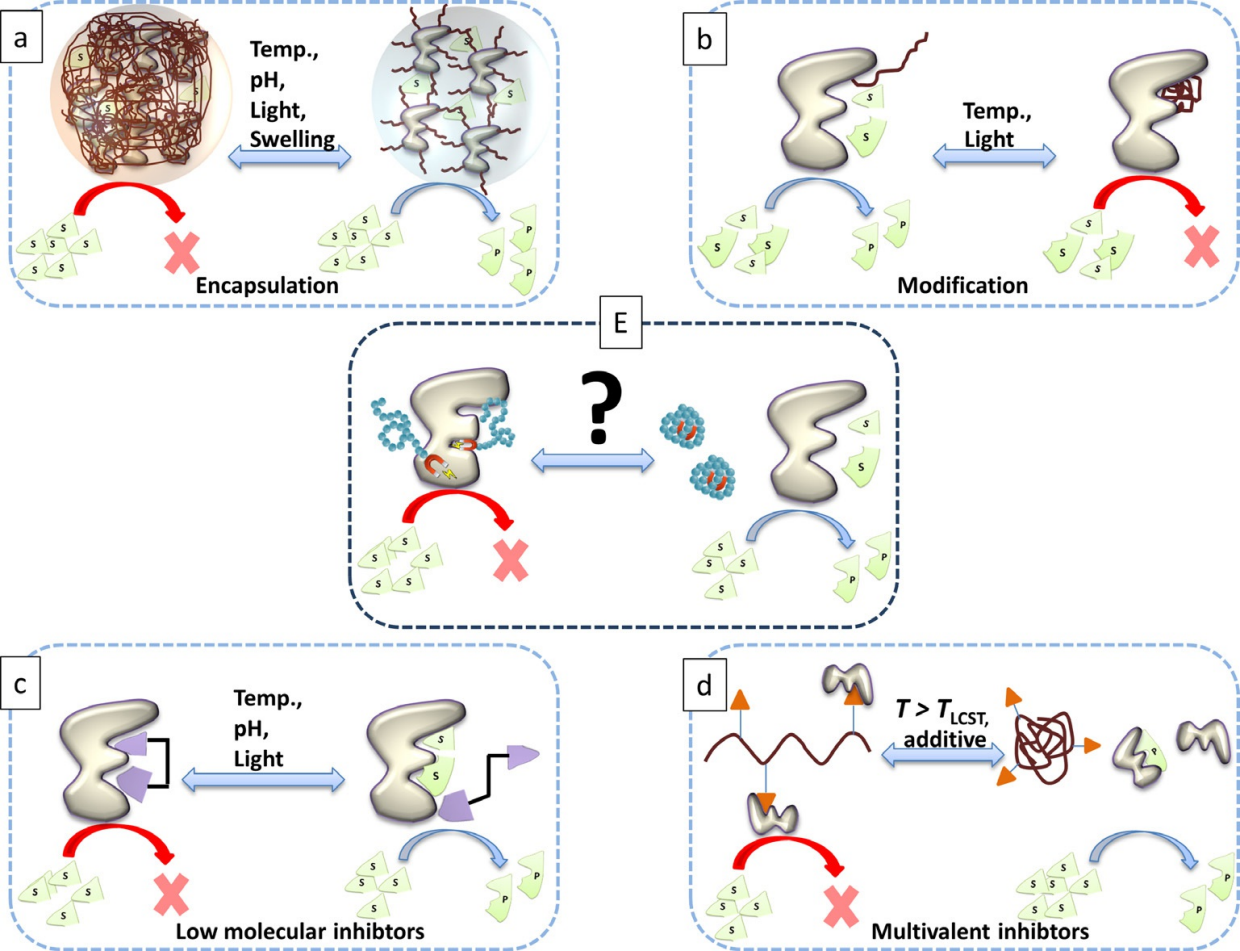
a)–d) Strategies for controlling enzyme activities found in the literature. e) Concept of the inhibition mechanisms for thermoresponsive poly‐2‐oxazoline polymers proposed in this work.

Typical strategies for controlling enzyme activity are shown in Figure 1.

The most common strategy in controlling the activity of biocatalysts is the encapsulation of enzymes into light‐ or thermoresponsive amphiphilic copolymers, hydrogels, polymersomes, or nanoparticles (Figure 1 a).^1^, ^2^, ^3^, ^4^ This way, the accessibility of the substrate is controlled by the switchable aggregation or swelling behavior of the surrounding shell. This works best for isolated enzymes and is usually not specific and only useful for larger substrates.

**Figure 2:**
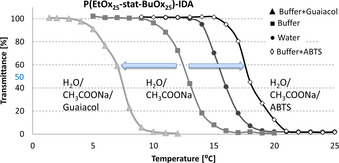
Turbidity curves of polymer P(EtOx_25_‐stat‐BuOx_25_)‐IDA (20 mg mL^−1^) in water, aqueous acetate buffer, acetate buffer + 8.26 mm guaiacol, and acetate buffer + 5 mm ABTS.

Another way to achieve this was pioneered by Stayton and Hoffmann in 2003, who have managed to control enzyme activity by attaching a thermosensitive copolymer based on *N*,*N*‐dimethylacrylamide (DMAM) and *N*‐4‐phenylazophenylacrylamide (AZAAm) monomers near the active site of the enzyme endoglucanase 12A (for the concept, see Figure 2 b, below). This conjugate showed 82 % of its original enzyme activity and a full activity shut off above the phase transition temperature of the UCST polymer.^5^ Others have successfully followed this approach by a number of further polymers attached to enzymes.^6^, ^7^, ^8^ Although elegant, creating such conjugates is still quite elaborate.

Alternatively, researchers have created low molecular weight enzyme inhibitors, which can be changed in their structure.^9^, ^10^ or form aggregates that are not active anymore.^11^ This method illustrated in Figure 1 c has the limitation that the change in structure is an equilibrium state not fully in favor of one or the other form and thus usually not efficient to fully switch enzyme activity.

The combination of polymers with enzyme inhibiting functional groups is a possibility to use the thermally induced phase transition of the latter with activity control of the biocatalysts (Figure 1 d). However, the enzyme inhibiting groups are still accessible after the phase transition resulting in no full switch ability.^12^

We propose that this can be circumvented by using enzyme inhibitors attached to the terminal of a thermosensitive polymer (Figure 1 E). A macromolecule designed this way should be able to “hide” the inhibitor inside the coil after thermally induced phase transition, which should enable a full on and off switching of the enzyme activity. Chang et al. could already show that the antibiotic ciprofloxacin, which is an enzyme inhibitor, could be thermally controlled in its activity by hindering the passage of the polymer through the bacterial cell membrane.^13^ So far it was not explored if the thermally induced phase transition can truly deactivate an inhibitor attached to the end group.

## Results and Discussion

We have previously reported on poly(2‐oxazoline)s with an iminodiacetic acid (IDA) end group (POx‐IDA) as an entropic, noncompetitive inhibitor for horse radish peroxidase (HRP), which is widely used in many biological assays, including ELISA, glucose sensors, hydrogen peroxide sensors.^14^, ^15^, ^16^, ^17^ These polymers were also found to be competitive inhibitors for laccase, which can be found in bioremediation,^18^ chemical synthesis,^19^ wine stabilization, and biosensing.^20^ Temperature control of the activity of these enzymes could improve the biosensors, but might also allow to specifically interact with production cascades to control the selective synthesis of fine chemicals.^21^, ^22^ Diluting the polymer/enzyme mixtures always results in full activity of the enzyme, indicating that the inhibition is fully reversible. Interestingly, POX‐IDAs are dead‐end inhibitors for both enzymes, that is, they can fully inhibit the enzyme activity. This offers the potential of fully switching enzyme activity. Poly(2‐oxazoline)s have been found to be excellent lower critical solution temperature (LCST) polymers, which can be adjusted in their cloud‐point temperature by copolymerization of different 2‐alkyl‐oxazolines and by varying the end groups.^23^, ^24^, ^25^

In order to render POx‐IDA into a thermoswitchable enzyme inhibitor, a number of different POx with LCST behavior was synthesized by polymerizing various 2‐alkyl‐2‐oxazolines as homopolymers and statistical copolymers that are known as LCST polymers from literature.^25^ The polymers where then terminated with dimethyl‐IDA (IDD) and subsequently treated with aqueous NaOH to achieve an IDA end group. All analytical data of these polymers including ^1^H NMR spectra and SEC results are given in Table S1 in the Supporting Information.

The thermoresponsive activity control was performed photometrically at temperatures below and above the cloud‐point temperature (*T*_cp_). Several substrates for HRP and laccase are known. Applying LCST polymers in a complex environment such as an enzyme assay or a chemical synthesis is always critical, because various factors such as cosolutes, buffer salts or any other chemical additive, for example, enzyme substrates, can interfere with *T*_cp_.^26^, ^27^ In order to determine if the polymers are affected by the contents of the respective enzyme assay solution, the cloud points were measured in assays for HRP and laccase activity using different substrates in the concentrations applied in the assay (Figure 2).

As seen in Figure 2 the *T*_cp_ of the investigated polymer of 17 °C in water downshifts by 2–3 K in the chaotropic acetate buffer. When adding ABTS (5 mm) to this buffer *T*_cp_ is increased by 5 K. In contrast, addition of the substrate guaiacol to the buffer affords a decrease in *T*_cp_ by 5–6 K. This shows a general problem of thermoresponsive polymers, which are greatly influenced by their surroundings and therefore become unreliable when used in changing natural environments. Table 1 summarizes the cloud‐point temperatures of the different POx‐IDAs in their respective enzyme assay.

**Table 1:** Cloud‐point temperatures *T*_CP_ of the synthesized POx with an IDA end group at 20 mg mL^−1^ in aqueous acetate buffer pH 5.0. The experimental protocol of the synthesis, the analytical data of the polymers, details on the *T*_CP_ measurements, the respective phase transition curves (Figures S1–S5), and the enzyme activity assay compositions are given in the Supporting Information.

	*T*_cp_ [°C]			
	buffer+	buffer+	buffer+	buffer+
	DMP	guaiacol	DMP off‐on	guaiacol off‐on
P(PropOx_55_)‐IDA	41	41	39–45	40–48
P(PropOx_14_‐stat‐iPropOx_25_)‐IDA	35	33	33–37	30–36
P(EtOx_26_‐stat‐BuOx_14_)‐IDA	20	19	13–25	12–26
P(EtOx_15_‐stat‐BuOx_15_)‐IDA	13	12	10–16	11–17
P(EtOx_25_‐stat‐BuOx_25_)‐IDA	7	8	4–10	5–11

[a] The measurements were carried out in 100 mm acetate buffer pH 5.0 with 2.8 mm DMP and 8.26 mm guaiacol, respectively. IDA is iminodiacetate, *T*_CP_ off=the highest temperature at which the transmittance is above 99 %, *T*_CP_ on=the lowest temperature at which the transmittance is below 1 %.

As seen in Table 1, the transition range of the POx‐IDA can be varied over a broad range of temperatures of *T*_cp on_ of 4–40 °C (full enzyme inhibition below these temperatures) and of *T*_cp off_ of 10–48 °C (full enzyme activity above these temperatures). The transition range of the investigated POx‐IDAs is rather broad compared the literature known systems, which is due to the relatively broad dispersity (Table S1) and the IDA end group. The statistical distribution of the different monomers in the copolymers leads to a further broadening of the transition. This will prevent a discrete switching but offers the possibility to gradually control (“dim”) the activity in a broad range with varying on and off regions ranging from below 4 to 48 °C.

The activity of laccase and HRP was measured in the presence of varying amounts of the respective POx‐IDA. The objective was to find a concentration where the enzyme activity is completely switched OFF (more than 99 % inhibition) at a certain temperature and fully switched ON at a higher temperature (more than 99 % activity). The activity was measured at concentrations of up to 8 mm POx‐IDA below the respective onset temperature *T*_cp on_. This concentration was found to be the highest value that allowed complete recovering of the enzyme activity upon heating above *T*_cp off_ for all POx‐IDA. The enzyme activities are always compared to the native enzyme activity at the respective temperature.

All copoly(2‐oxazoline)s P(EtOx‐stat‐BuOx)‐IDA fully inhibit both enzymes at 8 mm below *T*_cp on_ and the activity is fully recovered above the respective *T*_cp off_. In contrast, P(PropOx_55_)‐IDA fully inhibits HRP, but are is not capable of fully inhibit laccase at 8 mm (77 % inhibition). The copolymer P(PropOx_14_‐stat‐*i*ProOx_25_)‐IDA inhibits only laccase by more than 97 % a concentration of 8 mm, while it does not affect the activity of HRP at this concentration. This shows that not only the IDA group influences the inhibition, but also that changing the nature of the polymer backbone allows the creation of enzyme selective inhibitors.

**Figure 3:**
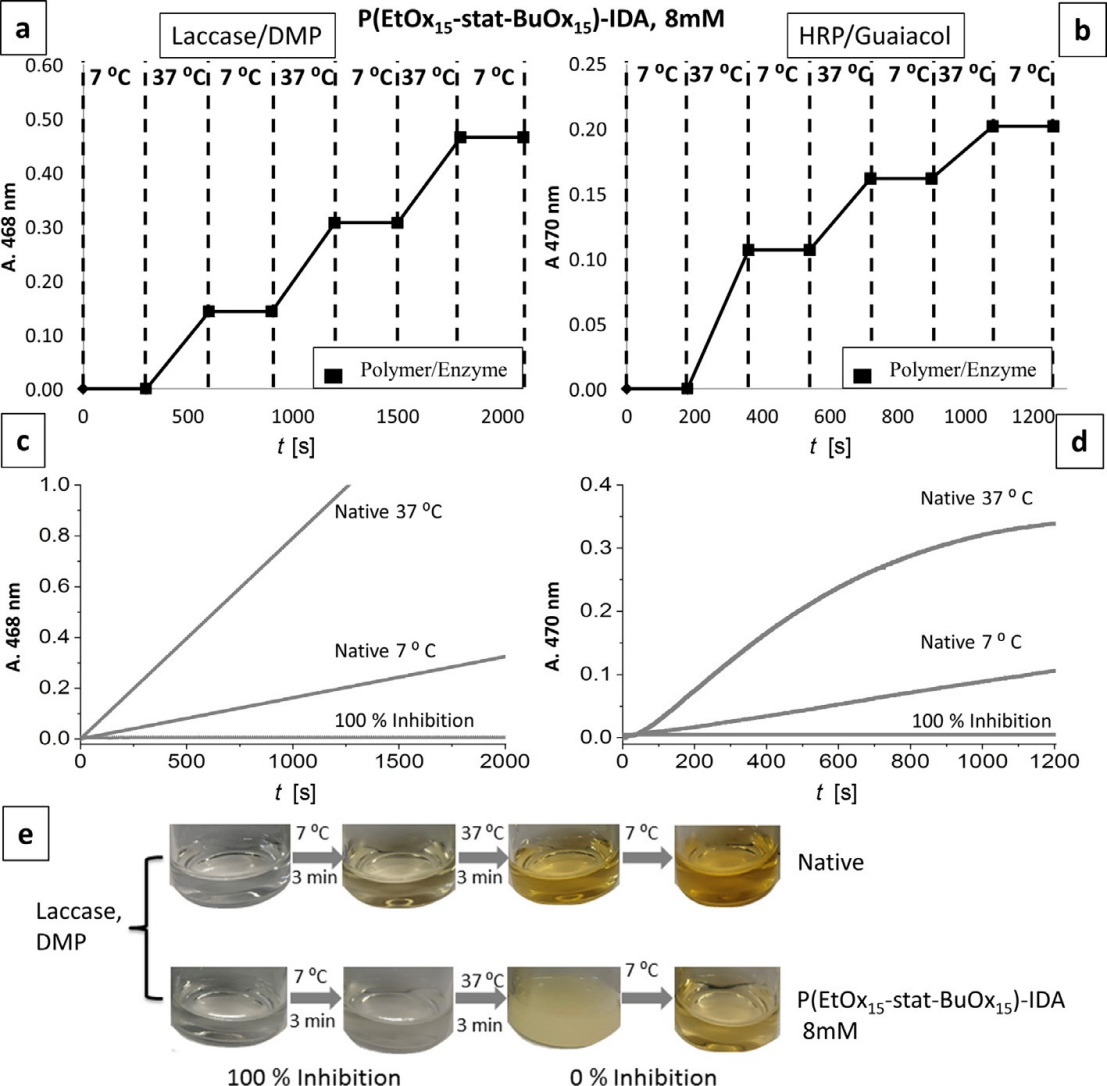
Increase in absorbance vs. time of a) laccase and b) HRP activity in an activity assay switched by P(EtOx_15_‐stat‐BuOx_15_)‐IDA under thermally cycling between 7 and 37 °C. Increase in absorbance vs. time of c) laccase and d) HRP in an assay without inhibitor at different concentrations. e) Photographs of the first thermal cycle of two laccase/DMP activity assays with and without the dead‐end inhibitor P(EtOx_15_‐stat‐BuOx_15_)‐IDA (8 mm).

Having established that POx‐IDAs are efficient thermal enzyme switches, it was now addressed whether this process is reversible. To this end the polymer P(EtOx_15_‐stat‐BuOx_15_)‐IDA and laccase or HRP, respectively, were added in a cuvette containing the respective activity assay and the absorbance was measured at 7 °C (below *T*_set off_) for 3 min. Then the cuvette was quickly heated to 37 °C (above *T*_set on_) using a water bath, was kept there for 2.5 min, and was then cooled to 7 °C using an ice bath and put back into the spectrophotometer. This cycle was repeated three times. As seen in Figure 3, the activity of the enzymes can indeed be reversibly switched ON and OFF with temperature. The switched ON laccase activity is always similar as proven by the equidistant absorbance increase. The activity of HRP seems to be lower with each activation step. This is due to the fact that HRP gets quickly deactivated in the activity assay as evident from the activity curve shown in Figure 3 d. Thus, the switching of the activity is fully reversible for both enzymes and the general activity of the respective enzyme is not affected by the switching.

While the change of temperature by 30 K slows the reaction rate of the laccase catalyzed oxidation by only a factor of 5 (due to the relatively low activation energy, Figure S11), the switchable inhibitor fully stops the reaction, which is a factor of at least 20 times more than the temperature effect. Figure 3 e shows photographs of the reaction mixtures of the first cycle that clearly illustrate the difference between inhibited and not inhibited reaction mixture.

**Figure 4:**
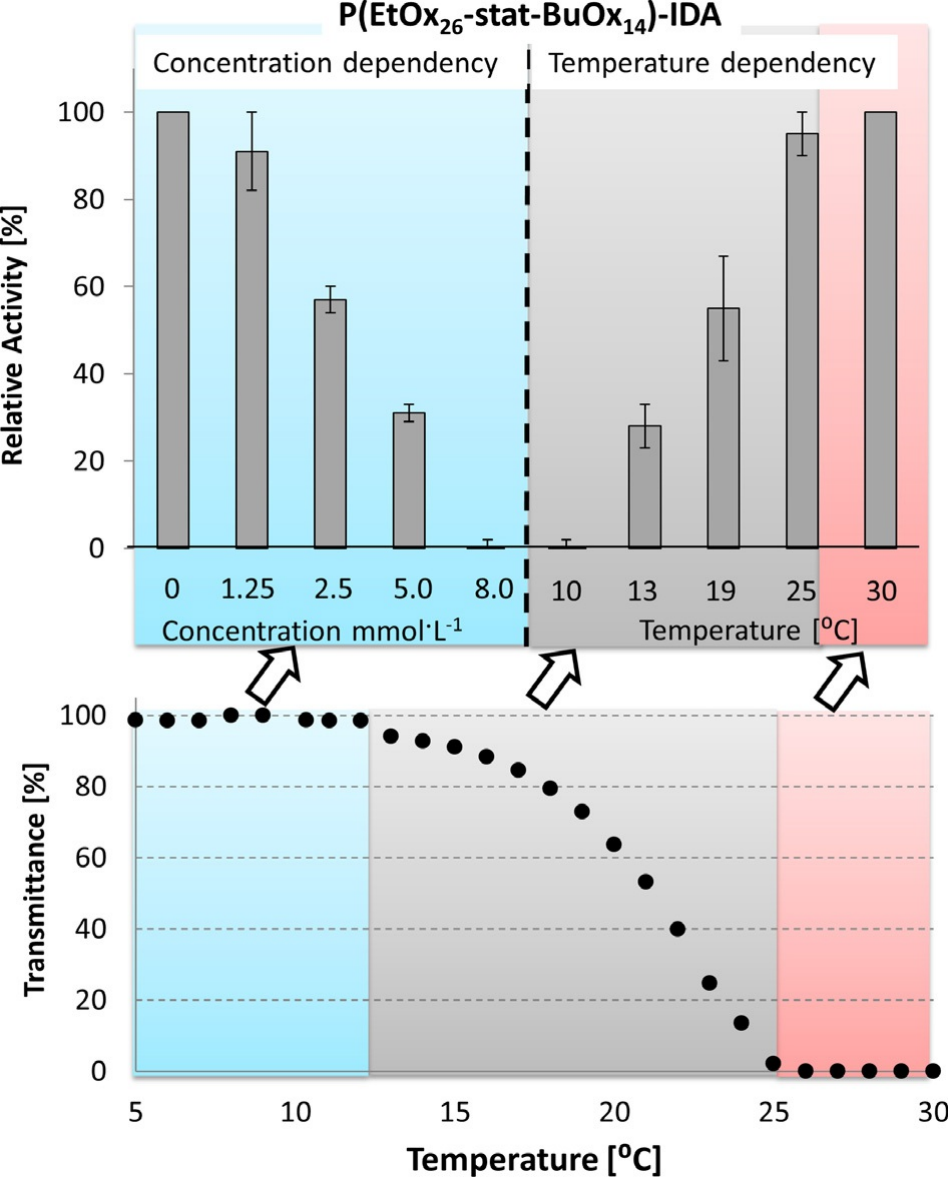
Top left: Change in activity of laccase at 10 °C as a function of the concentration of P(EtOx_26_‐stat‐BuOx_14_)‐IDA; Top right: Change in activity of laccase at different temperatures in the presence of 8 mm P(EtOx_26_‐stat‐BuOx_14_)‐IDA. Bottom: Turbidity curve of P(EtOx_26_‐stat‐BuOx_14_)‐IDA in aqueous acetate buffer (100 mm, pH 5.0) +2.8 mm DMP. The laccase assay was performed in acetate buffer with DMP as substrate. The activity was measured in triplicate, and the error bars show the standard deviation.

Besides switching enzyme activity on and off, the POx‐IDA are also capable of “dimming” enzyme activity. This is demonstrated on the example of P(EtOx_26_‐stat‐BuOx_14_)‐IDA that has a particularly broad transition range (*T*_cp_=20 °C, switching range 13–25 °C at 8 mm in the laccase assay). Figure 4 shows the relative activity of laccase in the presence of 8 mm P(EtOx_26_‐stat‐BuOx_14_)‐IDA at different temperatures compared with the respective thermal phase transition diagram of the polymer and the degree of inhibition at different concentrations at 10 °C. The results clearly prove that the laccase activity can be tuned by changing the temperature. For example, when the temperature was increased from 10 °C (activity <1 %) to 19 °C, the relative activity raises to 55 %. This corresponds to the inhibition of the enzyme by 2.5 mm the same polymer at 10 °C. In other words, some 70 % of the inhibitor is precipitated and thus inactive at 19 °C. Given the phase transition curve at the bottom of Figure 4, this is a likely scenario and supports the precipitation of the polymer as switching mechanism. The laccase activity is recovered to 100 % by further increasing the temperature to 30 °C.

The precipitation of the inhibitor does not only lead to activation of the enzyme, it also offers the possibility to remove it. This was tested on the example of P(EtOx_26_‐stat‐BuOx_14_)‐IDA, which was added to a laccase solution (final concentration of 8 mm) and cooled to 7 °C, which results in full inhibition of the enzyme. Then the temperature was increased to 37 °C and the precipitate was filtered off using a polypropylene syringe filter. The resulting filtrate showed 92 % of the activity of the respective laccase control solution, indicating that the switchable inhibitor can easily be removed in contrast to systems, where the enzyme is covalently modified with thermoresponsive polymer.

## Conclusions

We have shown that thermoresponsive poly(2‐oxazoline)s terminated with one IDA group are dead‐end inhibitors for laccase and HRP. Increasing the temperature results in full recovery of the activity of both enzymes. The process is fully reversible and does not interfere with the protein′s activity. This complete switching can be used to fully thermally control the bioactivity of these widely used, important enzymes. Biocompatible poly(2‐oxazoline)s, which have been shown to be suitable for the conjugation of various drugs and proteins,^28^, ^29^ have already been successfully used to conjugate inhibitors for collagenases,^30^ d‐alanine transpeptidase,^31^ and gyrase,^32^, ^33^ which are highly relevant for biomedical applications. Therefore, the approach presented here will likely be applicable to a broad range of temperature‐controllable enzymatic processes. The selectivity of some of the inhibitors shown here might be used to temperature control the activity of enzymes in multienzyme systems.

## Conflict of interest

The authors declare no conflict of interest.

## Supporting information

As a service to our authors and readers, this journal provides supporting information supplied by the authors. Such materials are peer reviewed and may be re‐organized for online delivery, but are not copy‐edited or typeset. Technical support issues arising from supporting information (other than missing files) should be addressed to the authors.

Supplementary
