# Supplementary material for: Full Thermal Switching of Enzymes by Thermoresponsive Poly(2‐oxazoline)‐Based Enzyme Inhibitors
Source: Chemistry. 2020 Sep 23;26(59):13367–71. doi: 10.1002/chem.202001909 (PMC7702056; doi:10.1002/chem.202001909)
Supplement: Supplementary file 1 — Supplementary [file CHEM-26-13367-s001.pdf]

Chemistry–A European Journal

Supporting Information

Full Thermal Switching of Enzymes by Thermoresponsive Poly(2-oxazoline)-Based Enzyme Inhibitors

Montasser Hijazi, Esra Türkmen, and Joerg C. Tiller*[a]

Experimental

Instruments

^{1}H NMR spectra were recorded in $CDCl_3$-d1 using a Nanobay AVANCE-III HD-400 spectrometer with a 5 mm BBFOsmart probe from Bruker BioSpin GmbH operating at 400 MHz and on a DD2-500 spectrometer with 5 mm triple resonance H(C,X) probe from Agilent Technologies operating at 500 MHz.

Ultraviolet-visible spectroscopy (UV-VIS) on a double-beam photometer was used for the monitoring of the enzyme activity using an Analytik Jena Specord 210 spectrophotometer.

Size exclusion chromatography (SEC) was performed on a Viscotek GPCMax equipped with a refractive index (RI) detector (tempered to 55°C) using a Tosoh TSKgel GMHHR-M (5.0 µm pores, 2x + 1x precolumn) column set. As eluent, saline N,N-dimethylformamide (DMF+LiBr, 20 mmol) was used at 60°C at a flow rate of 0.70 mL·min-1. Calibration was performed with poly(styrene) standards (from Viscotek).

All polymerizations were performed using a microwave-assisted synthesizer from CEM with a vertically focused IR sensor.

Methods

Material: All chemicals and solvents were purchased from Acros, Merck, Fluka and Sigma Aldrich. Peroxidase from horseradish (EC 1.11.1.7) and laccase from versicolor trametes were purchased from Sigma Aldrich. 1,3-dimethoxy-2-hydroxybenzene (DMP) and *o*-methoxyphenol (Guaiacol) were purchased from Acros, [2,2-azino-bis(3-ethylbenzothiazoline-6-sulfonic acid)] diammonium salt (ABTS) was purchased from Sigma-Aldrich.

Synthesis of 2-R-2-oxazoline

The syntheses of the HepOx, BuOx, and iPrOx were carried out according to literature.[1] [2]

Synthesis of Poly(2-oxazoline)-IDA

The syntheses of the homo and co-polymers terminated with IDA were carried out according to literature.[3] [4] [5] The composition of the polymers was calculated from ^{1}HNMR spectrum in $CDCl_3$.

Table S1: analytical data of the synthesized POx. The initiator for POx-IDA was methyl tosylate. The termination was performed with 2.5 eq. dimethyl 2,2'-azanediyldiacetate. F_d is the degree of termination, H_d is the degree of hydrolysis

**Polymer designations**	**M_{NMR} g mol^{-1}**	**M_{GPC} g mol^{-1}**	**F_d %**	**H_d %$^{a)}$**	**PDI**
P(PropOx$_{55}$)-IDD	6300	4000	100	100	1.15
P(PropOx$_{14}$-stat-iPropOx$_{25}$)-IDD	5000	3100	96	100	1.44
P(EtOx$_{26}$-stat-BuOx$_{14}$)-IDD	4500	4100	100	100	1.30
P(EtOx$_{15}$-stat-BuOx$_{15}$)-IDD	3500	4200	100	100	1.37
P(EtOx$_{25}$-stat-BuOx$_{25}$)-IDD	4300	3500	100	100	1.29

IDD: Dimethyl 2,2'-iminodiacetate

<u>NMR spectra and ESC traces</u>

P(PropOx$_{55}$)-IDD

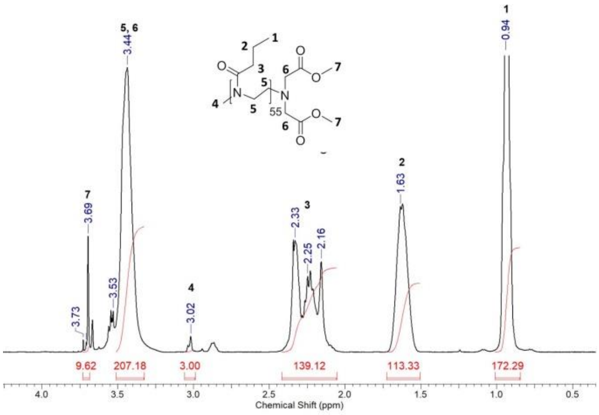

P(PropOx$_{55}$)-IDA

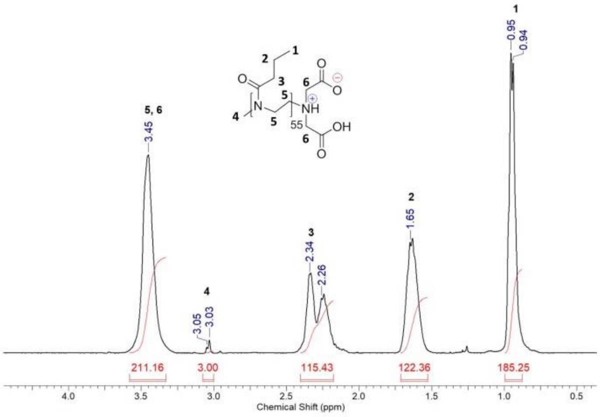

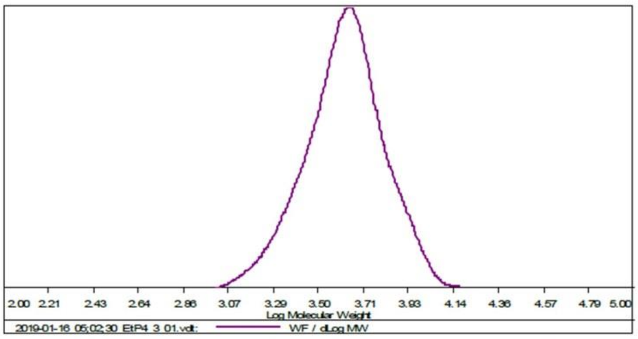

Sample	Mn	Mw	Mz	IV	Rh
2019-01-16_05;02;30_EtP4_3_01.vdt	3.964	4.730	5.563	0.0000	0.00

Figure S1: above, ^{1}H NMR spectra of the polymer P(PropOx$_{55}$)-IDD before and after alkaline hydrolysis with 0.025 M NaOH at 50 ^{o}C. Below, the SEC trace for the P(PropOx5$_5$) precursor.

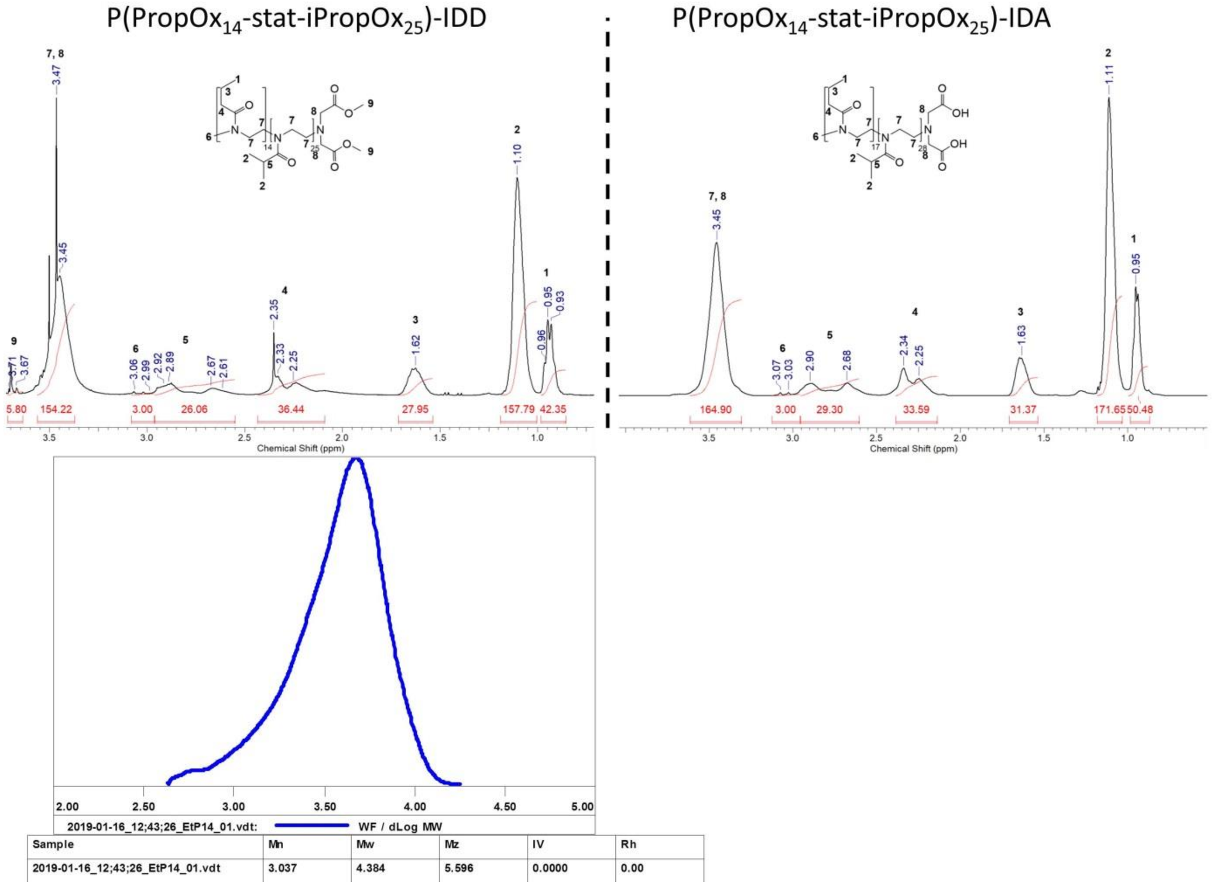

Sample	Mn	Mw	Mz	IV	Rh
2019-01-16_12;43;26_EtP14_01.vdt	3.037	4.384	5.596	0.0000	0.00

Figure S2: above, ^{1}H NMR spectra of the polymer P(EtOx$_{14}$-stat-BuOx$_{25}$)-IDD before and after alkaline hydrolysis with 0.025 M NaOH at 50 ^{o}C. Below, the SEC trace for the P(EtOx$_{14}$-stat-BuOx$_{25}$) precursor.

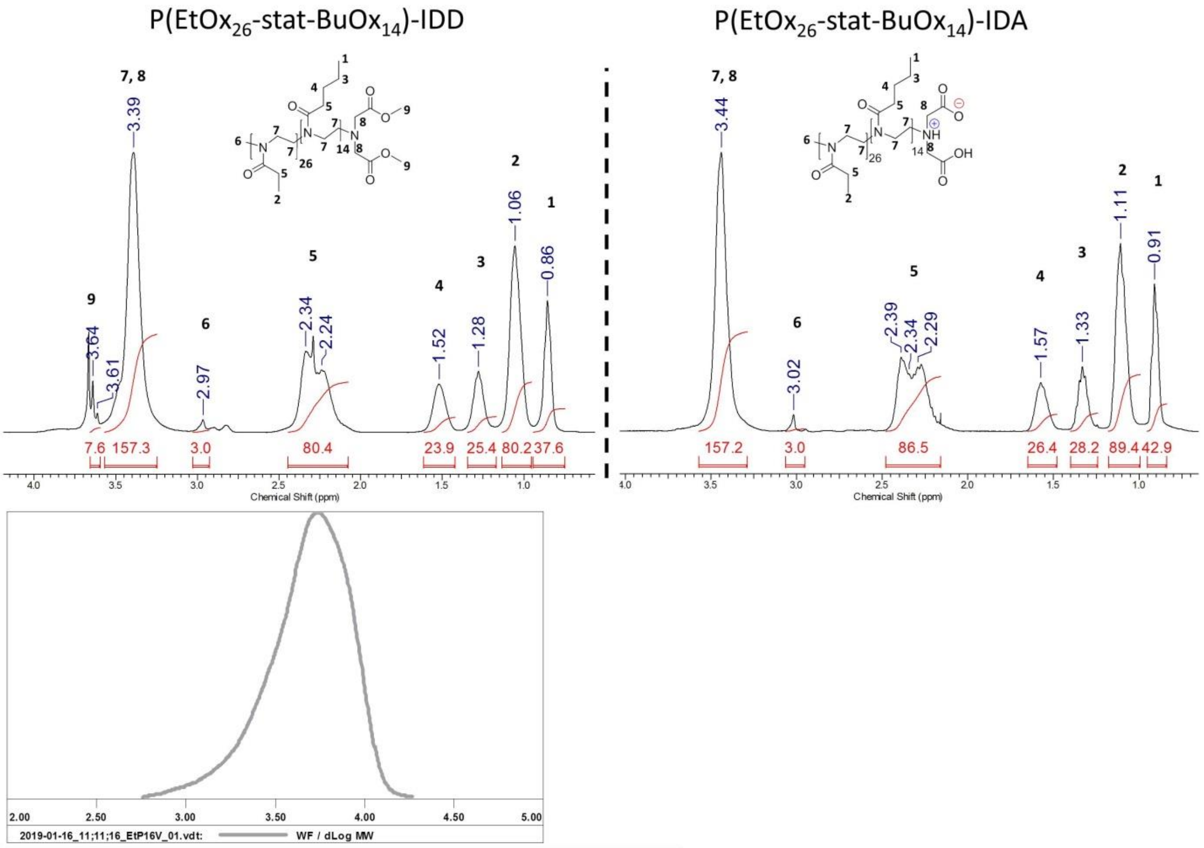

Sample	Mn	Mw	Mz	IV	Rh
2019-01-16_11;11;16_EtP16V_01.vdt	4.163	5.492	6.666	0.0000	0.00

Figure S3: above, ^{1}H NMR spectra of the polymer P(EtOx$_{26}$-stat-BuOx$_{14}$)-IDD before and after alkaline hydrolysis with 0.025 M NaOH at 50 ^{o}C. Below, the SEC trace for the P(EtOx$_{26}$-stat-BuOx$_{14}$) precursor.

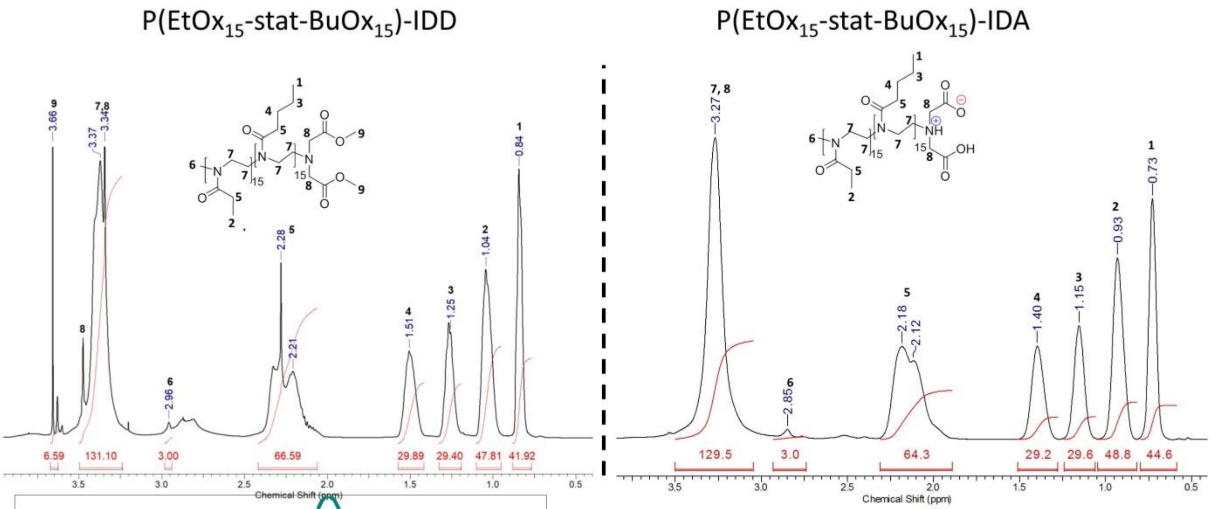

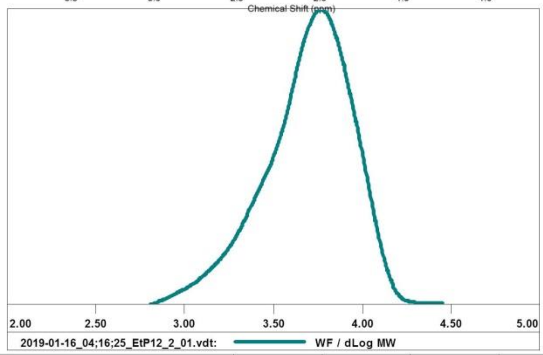

Sample	Mn	Mw	Mz	IV	Rh
2019-01-16_04;16;25_EtP12_2_01.vdt	4.185	5.754	7.239	0.0000	0.00

Figure S4: above, ^{1}H NMR spectra of the polymer P($EtOx_{15}$-stat-$BuOx_{15}$)-IDD before and after alkaline hydrolysis with 0.025 M NaOH at 50 ^{o}C. Below, the SEC trace for the P($EtOx_{15}$-stat-$BuOx_{15}$) precursor.

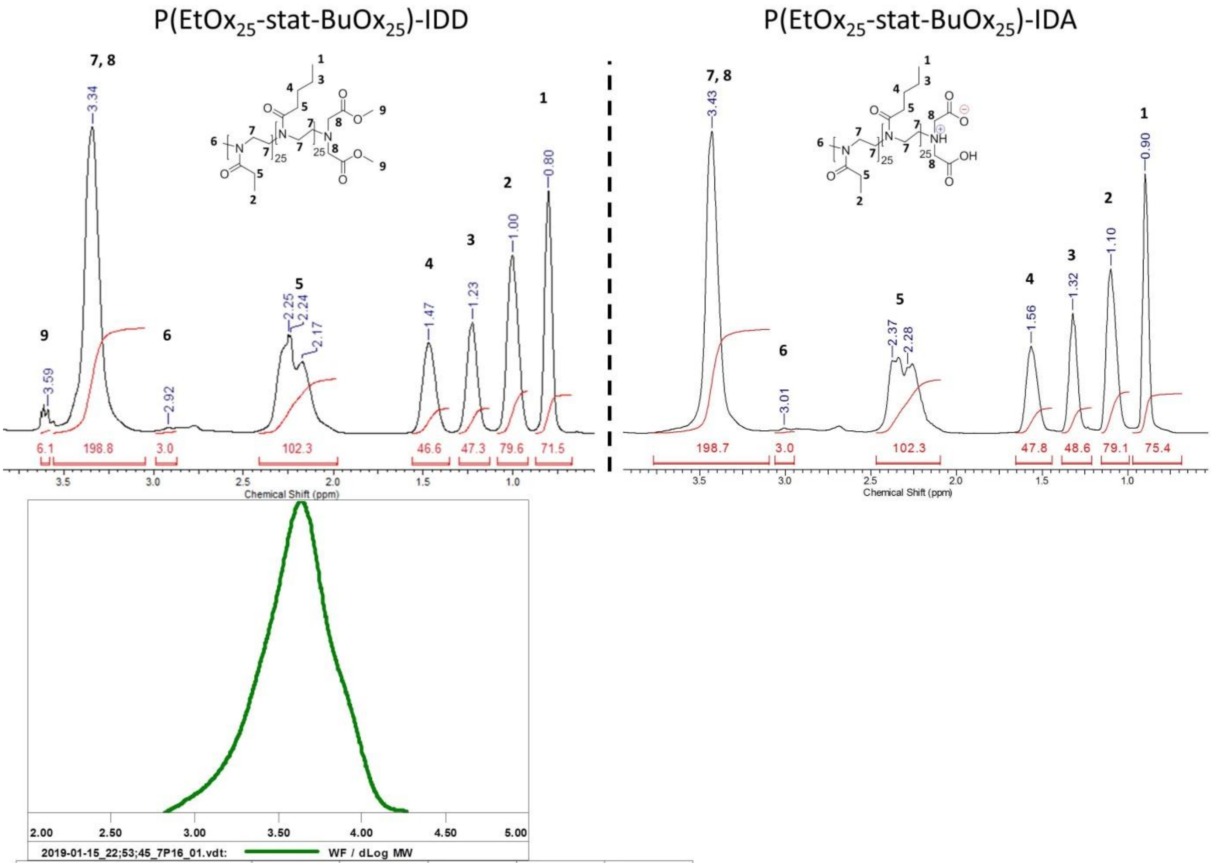

Sample	Mn	Mw	Mz	IV	Rh
2019-01-15_22;53;45_7P16_01.vdt	3.519	4.589	5.734	0.0000	0.00

Figure S5: above, ^{1}H NMR spectra of the polymer P(EtOx$_{25}$-stat-BuOx$_{25}$)-IDD before and after alkaline hydrolysis with 0.025 M NaOH at 50 ^{0}C. Below, the SEC trace for the P(EtOx$_{25}$-stat-BuOx$_{25}$) precursor.

Determination of cloud points

The polymer sample was dissolved in water or in buffer at 8 mM polymer concentration. The aqueous polymer solution was then transferred into a quarz-glass cuvette and placed in the photometer (Specord® S 600 from ANALYTIK JENA) and the transmission was measured. A temperature ramp from 0-70 °C was applied using peltier element with a heating rate of 30 K/h. The temperature at which the transmission reached 50% was considered as cloud point (T_{cp}).

Laccase-assay with DMP substrate: The laccase activity in presence and absence of POx was determined using 2.8 mM of 2,6-dimethoxyphenol (DMP) substrate in 100 mM acetate buffer pH 5.[6] The measurement was carried out in thin cuvette (750 µl, 0.2 cm) in tempered photometer. Different concentration of POx in the range from 1.25-8 mM was dissolved in 500 µl of acetate buffer. Then, 143 µl enzyme solutions (0.02 mg/ml) mixed to the polymer buffer mixture and the temperature was desired below or above T_{cp} (according to the T_{cp} values in table1). Subsequently, 71 µl of 28 mM DMP solution was added and the increase of absorbance was determined at a wavelength of 468 nm for 3 min.

Peroxidase-assay with Guaiacol substrate: the peroxidase activity in presence and absence of POx was determined using Guaiacol and hydrogen peroxide substrate in 100 mM acetate buffer at pH 5. Firstly, different concentration of polymer solutions (1.25- 8 mM) was dissolved in 8.26 mM Guajacol buffered solution. In 0.2 cm thin cuvette, 11.5 µl peroxidase (0.05 mg/ml) was mixed to 680 µl polymer substrate mixtures. The desired temperature was set in tempered photometer. As soon as the absorbance remained constant, 24 µl of H_2O_2 (0.3 wt.%) was added and the increase of absorbance was photometrically determined at wavelength of 470 nm for 3 min.

Activity switch measurements:

The activity of laccase and peroxidase were tested according to the measurements above with P(EtOx$_{15}$-stat-BuOx$_{15}$)-IDA. The polymer and the enzymes laccase or HRP, respectively, were added in a cuvette containing the respective activity assay and the absorbance was measured at 7°C (below T_{cp}) for 3 min. Then cuvette was quickly heated to 37°C (above $T_{set\ off}$) using a warm water bath, was kept there for 2 min 30s, and was then cooled below to 7

°C using an ice bath and put back into the spectrophotomer. This cycle was repeated three times.

Determination of *Ea* for laccase from trametes versicolor: enzyme solution was prepared with laccase from trametes versicolor $2.2*10^{-3}$ mg/ml dissolved in in 100 mM sodium acetate (pH 5). A substrate solution was composed of 28 mM and 100 mM sodium acetate buffer (pH 5). Assay was initiated by mixing 800 µl acetate buffers, 100 µl of enzyme and 100 µl of DMP at the respective temperatures 10, 13, 19, 25 and 37 ^{o}C. The activity was reported to follow a zero-order reaction $X = k{\cdot}t$ where X is the absorbance measured at 468 nm, k is the reaction rate constant and t is the reaction time. K was determined by the slope of the line created by plotting the reaction time against absorbance. The activation energy was appreciated by the Arrhenius equation expressed in logarithmic term Eq 1.

$$\mathrm{Ln}k=\ln A + \{(-Ea)/R\}*(1/T)$$

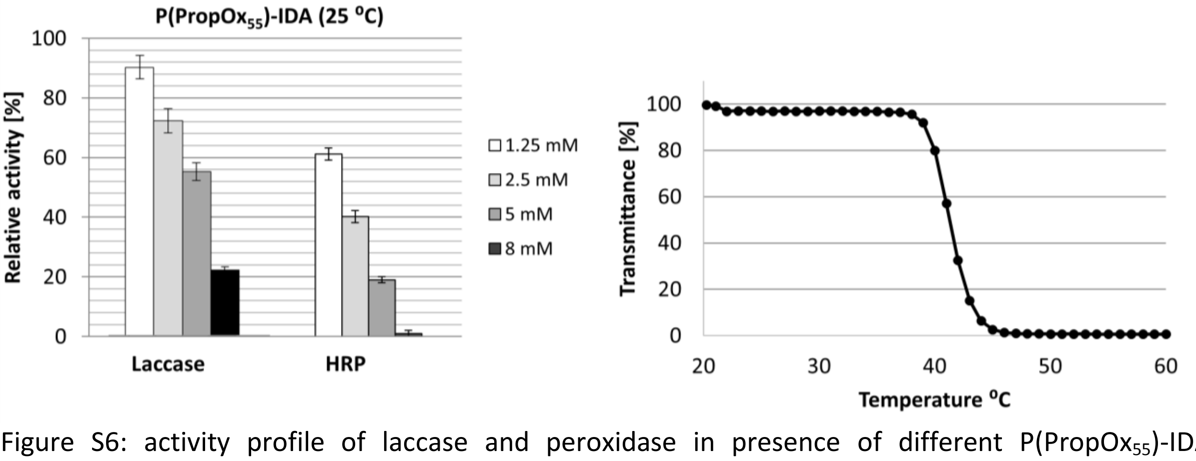

Figure S6: activity profile of laccase and peroxidase in presence of different P(PropOx$_{55}$)-IDA concentrations at 25 ^{o}C (left), the cloud points were performed in aqueous mixture of acetate buffer 100 mM and DMP 2.8 mM (right).

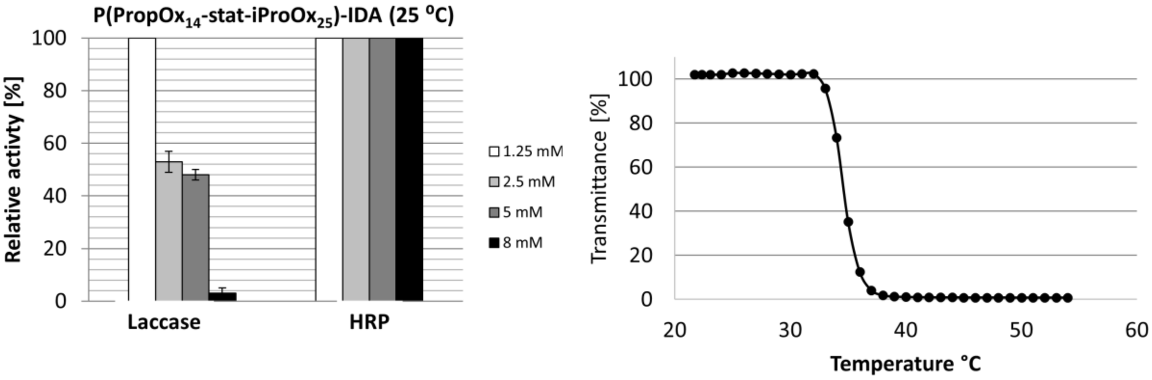

Figure S7: activity profile of laccase and peroxidase in presence of different P(PropOx$_{14}$-stat-iProOx$_{25}$)-IDA concentrations at 25 ^{o}C (left), the cloud points were performed in aqueous mixture of acetate buffer 100 mM and DMP 2.8 mM (right).

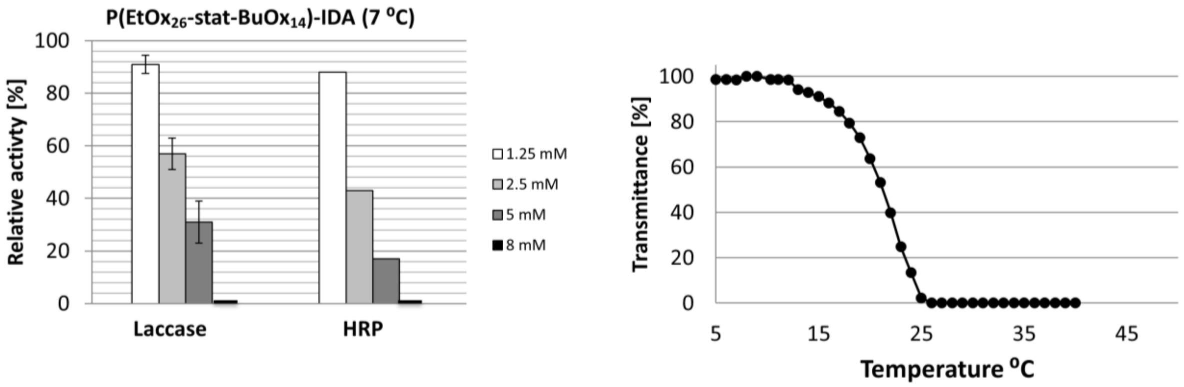

Figure S8: activity profile of laccase and peroxidase in presence of different P(EtOx$_{26}$-stat-BuOx$_{14}$)-IDA concentrations at 7 ^{o}C (left), the cloud points were performed in aqueous mixture of acetate buffer 100 mM and DMP 2.8 mM (right).

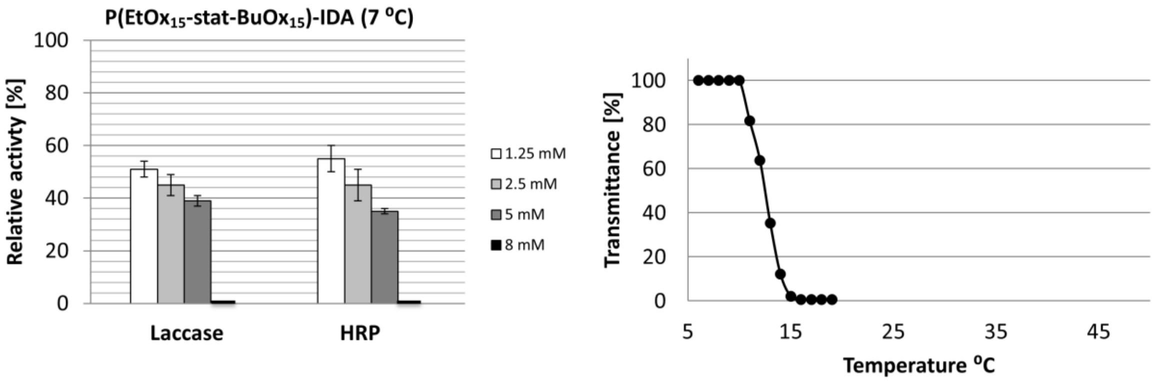

Figure S9: activity profile of laccase and peroxidase in presence of different P($EtOx_{15}$-stat-$BuOx_{15}$)-IDA concentrations at 7 ^{o}C (left), the cloud points were performed in aqueous mixture of acetate buffer 100 mM and DMP 2.8 mM (right).

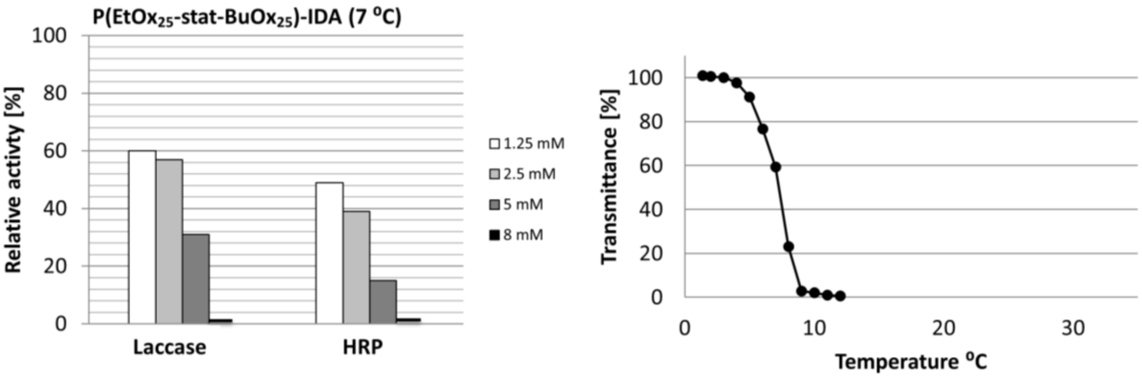

Figure S10: activity profile of laccase and peroxidase in presence of different P($EtOx_{21}$-stat-$BuOx_{18}$)-IDA concentrations o at 7 ^{o}C (left), the cloud points were performed in aqueous mixture of acetate buffer 100 mM and DMP 2.8 mM (right).

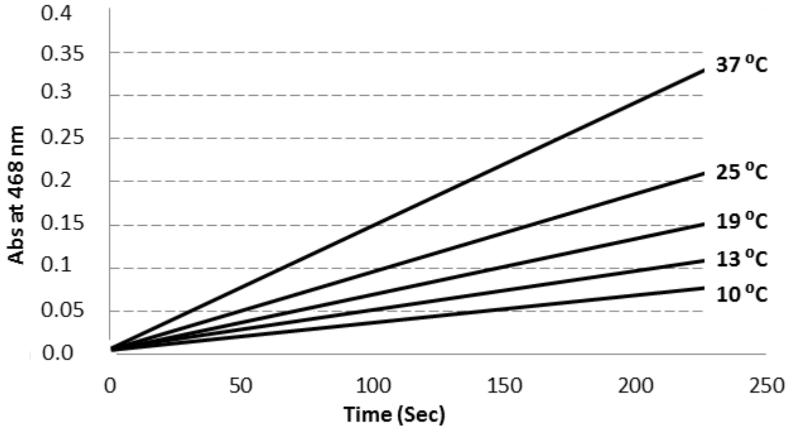

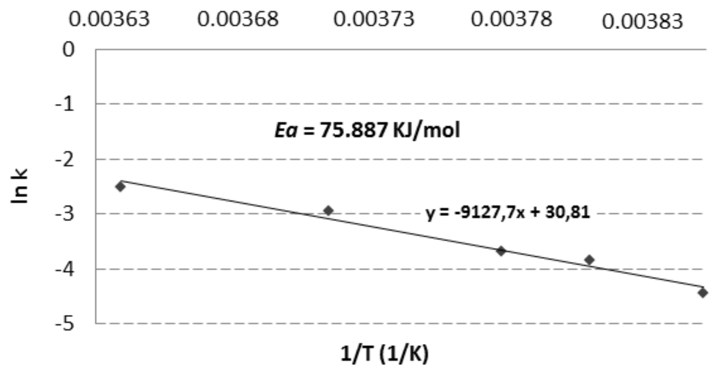

Figure S11: Temperature dependency of laccase activity for the oxidation of DMP with oxygen (above). Linearized Arrhenius plot of ln k vs. 1/T of the reactions rates determined from the curves in diagram (below).

References:

[1] C. Krumm, S. Konieczny, G. J. Dropalla, M. Milbradt, J. C. Tiller, *Macromolecules* **2013**, *46*, 3234-3245.

[2] M. Schmidt, T. Raidt, S. Ring, S. Gielke, C. Gramse, S. Wilhelm, F. Katzenberg, C. Krumm, J. C. Tiller, *European Polymer Journal* **2017**, *88*, 562-574.

[3] M. Hijazi, M. Schmidt, H. Xia, J. Storkmann, R. Plothe, D. D. Santos, U. Bednarzick, C. Krumm, J. C. Tiller, *Polymer* **2019**, *175*, 294-301.

[4] M. Hijazi, C. Krumm, S. Cinar, L. Arns, W. Alachraf, W. Hiller, W. Schrader, R. Winter, C. Tiller Joerg, *Chemistry – A European Journal* **2018**, *24*, 4523-4527.

[5] M. Hijazi, P. Spiekermann, C. Krumm, J. C. Tiller, *Biotechnology and Bioengineering* **2019**, *116*, 272-282.

[6] A. Paszczyński, R. L. Crawford, V.-B. Huynh, in *Methods in Enzymology, Vol. 161*, Academic Press, **1988**, pp. 264-270.
